# Safety Assessment of Dangerous Goods Transport Enterprise Based on the Relative Entropy Aggregation in Group Decision Making Model

**DOI:** 10.1155/2014/571058

**Published:** 2014-11-05

**Authors:** Jun Wu, Chengbing Li, Yueying Huo

**Affiliations:** School of Transportation, Inner Mongolia University, Hohhot, Inner Mongolia 010070, China

## Abstract

Safety of dangerous goods transport is directly related to the operation safety of dangerous goods transport enterprise. Aiming at the problem of the high accident rate and large harm in dangerous goods logistics transportation, this paper took the group decision making problem based on integration and coordination thought into a multiagent multiobjective group decision making problem; a secondary decision model was established and applied to the safety assessment of dangerous goods transport enterprise. First of all, we used dynamic multivalue background and entropy theory building the first level multiobjective decision model. Secondly, experts were to empower according to the principle of clustering analysis, and combining with the relative entropy theory to establish a secondary rally optimization model based on relative entropy in group decision making, and discuss the solution of the model. Then, after investigation and analysis, we establish the dangerous goods transport enterprise safety evaluation index system. Finally, case analysis to five dangerous goods transport enterprises in the Inner Mongolia Autonomous Region validates the feasibility and effectiveness of this model for dangerous goods transport enterprise recognition, which provides vital decision making basis for recognizing the dangerous goods transport enterprises.

## 1. Introduction

With the rapid development of society and continuous improvement of domestic economy in our nation, the occupations of the dangerous goods transport accounted in the entire transport system have been constantly raised. The dangerous goods refer to a kind of materials and goods which is flammable, explosive, toxic, strongly corrosive and heavily radioactive, and so forth [1]. Any links may easily cause accidents which can endanger people's life and property and pollute the environment in the process of transportation. Just for these special characteristics, it is extremely important to make safety assessment of dangerous goods transport enterprise.

At present, there are few available studies on safety assessment of dangerous goods transport enterprise in our country. For instance, Xiu and Zhang established the evaluation index system and fuzzy synthetic evaluation model used for safety management of dangerous goods transport and then assessed the safety of dangerous goods transport enterprise [2]. Huo et al. applied Likert-type scale based on Matter Element Analysis theory to assess the safety of dangerous goods transport enterprise [3]. While Yan and his partners integrated the Fuzzy Decision theory, group decision making, and TOPSIS, put forward a method to make safety assessment of dangerous goods transport enterprise based on Fuzzy TOPSIS [4]. In foreign countries, they focused on the dangerous goods transport routes optimization, risk assessment, emergency tube principle, and the development of decision support system research [5–9].

Above studies have provided a theoretical reference to assess the safety of dangerous goods transport enterprise, but they still need further improvement in aspects of index value and weight assignment. For instance, a certain index value in index system of safety assessment of dangerous goods transport enterprise may change with the passage of time, change of environment, affection of inner or outer factors, and shift of personal subjective wishes. It will not be accurate enough on assessment result in certain degree if they are assessed as static indexes. Moreover, because of the existing diversity on knowledge, experience, and preference among the experts, giving the same weight value may not be objective. Therefore, this paper researches on the safety assessment of dangerous goods transport enterprise using optimization model based on relative entropy in group decision making.

## 2. The Safety Assessment Model of Multiobjective Dangerous Goods Transport Enterprise Based on Entropy

Suppose *A* = {*a*_*j*_,  *j* = 1,2,…, *n*} is a dangerous goods transport enterprise set to be assessed, wherein *a*_*j*_ is the enterprise *j*; and *B* = {*b*_*i*_,  *i* = 1,2,…, *m*} is a set of assessment indexes from experts, wherein *b*_*i*_ represents index *i*. Namely, there are total *n* experts to make an assessment on *m* indexes in an assessment program. According to dynamic multivalue background, we adopt the following steps to establish a safety assessment model of multiobjective dangerous goods transport enterprise based on entropy.

*Step 1*. Identify the dynamic indexes and transform to static ones.

Firstly, analyse the attribute of safety assessment indexes on dangerous goods transport enterprise and identify the dynamic indexes. Then treat them statically according to the way described in [10], as showed in the following.

(1) According to the principle combining with qualitative and quantitative, the dynamic index's attribute value recorded for *k* times in different periods is defined as follows:
  (1) $$\begin{array}{c} M^{\left(k\right)}={\left\{m_{1}^{\left(k\right)},m_{2}^{\left(k\right)},m_{3}^{\left(k\right)},\ldots ,m_{n}^{\left(k\right)}\right\}}^{T}\;\left(k=1,2,\ldots \right). \end{array}$$
  And the weight vector and weight vector set of corresponding index in different period are given as follows:
  (2) $$\begin{array}{c} u^{\left(k\right)}=\left(u_{1}^{\left(k\right)},u_{2}^{\left(k\right)},\ldots ,u_{n}^{\left(k\right)}\right)\in U^{\left(k\right)},\\ U^{\left(k\right)}=\left\{\left(u_{1}^{\left(k\right)},u_{2}^{\left(k\right)},\ldots ,u_{n}^{\left(k\right)}\right)\;|\;\overset{n}{\underset{j=1}{\sum }}u_{j}^{\left(k\right)}=1,\;\;k=1,2,\ldots \right\}. \end{array}$$
(2) Calculate static value of all dynamic indexes using the following formula:
  (3) M=mj1+∑k=2ujkΔmjk ∣ Δmjk=mjk−mjk−1, k=1,2,…;j=1,2,…,n.

*Step 2.* Calculate multi-index assessment matrix as follows:

(4) $$\begin{array}{c} B^{\prime }=\left[\begin{array}{cccc} b_{11}^{\prime } & b_{12}^{\prime } & \cdots & b_{1n}^{\prime }\\ b_{21}^{\prime } & b_{22}^{\prime } & \cdots & b_{2n}^{\prime }\\ \vdots & \vdots & \cdots & \vdots \\ b_{m1}^{\prime } & b_{m2}^{\prime } & \cdots & b_{mn}^{\prime } \end{array}\right], \end{array}$$

where *b*_*ij*_′ is the weight of index *i* given by expert *j*; standardize *B*′, and then we get *B* = (*b*_*ij*_)_*m*×*n*_, and *b*_*ij*_ ∈ [0,1]; the value of *b*_*ij*_ depends on the following situations.

If the situation becomes better when the value of *b*_*ij*_ is median, then:

(5) $$\begin{array}{c} b_{ij}=\frac{2\left|\left(\left(\max_{j}\left\{b_{ij}^{\prime }\right\}-\min_{j}\left\{b_{ij}^{\prime }\right\}\right)/2\right)-b_{ij}^{\prime }\right|}{\max_{j}\left\{b_{ij}^{\prime }\right\}-\min_{j}\left\{b_{ij}^{\prime }\right\}}. \end{array}$$

If the situation is better when the value of *b*_*ij*_ becomes bigger, then:

(6) $$\begin{array}{c} b_{ij}=\frac{b_{ij}^{\prime }-\min_{j}\left\{b_{ij}^{\prime }\right\}}{\max_{j}\left\{b_{ij}^{\prime }\right\}-\min_{j}\left\{b_{ij}^{\prime }\right\}}. \end{array}$$

If the situation is better when the value of *b*_*ij*_ becomes smaller, then:

(7) $$\begin{array}{c} b_{ij}=\frac{\max_{j}\left\{b_{ij}^{\prime }\right\}-b_{ij}^{\prime }}{\max_{j}\left\{b_{ij}^{\prime }\right\}-\min_{j}\left\{b_{ij}^{\prime }\right\}}. \end{array}$$

*Step 3.* Define the entropy weight of every assessment index according to the following method.

(1) Among assessment of indexes with experts, the entropy of index is defined as follows:
  (8) $$\begin{array}{c} H_{i}=\frac{-1}{\ln n}\overset{n}{\underset{j=1}{\sum }}f_{ij}\ln f_{ij}\;\left(i=1,2,\ldots ,m\right), \end{array}$$
  where*f*_*ij*_ = *b*_*ij*_/∑_*j*=1_^*n*^ *b*_*ij*_. Note that ln⁡*f*_*ij*_ has no sense when *f*_*ij*_ = 0, thus defining *f*_*ij*_ as *f*_*ij*_ = (1 + *b*_*ij*_)/(1 + ∑_*j*=1_^*n*^ *b*_*ij*_).
(2) Calculate entropy weight of every assessment index in expression of *W*_*j*_ = (*λ*_*i*_)_1×*m*_, wherein *λ*_*i*_ = (1 − *H*_*i*_)/(*m* − ∑_*i*=1_^*m*^ *H*_*i*_), and ∑_*i*=1_^*m*^ *λ*_*i*_ = 1.

*Step 4*. Identify positive ideal point and negative ideal point.

After getting entropy weight, we can introduce *λ*_*i*_ into standardized matrix *B*′ and then get normalized matrix: *B* ^*^ = (*b*_ij_^*^)_*m*×*n*_, wherein *b*_ij_^*^ = *λ*_*i*_ *b*_*ij*_.

Thus positive ideal point and *nP* ^+^ = (*p*_1_^+^, *p*_2_^+^,…, *p*_*m*_^+^)^*T*^ negative ideal point, *P* ^+^ and *P* ^−^, respectively, can be expressed as follows:

(9) $$\begin{array}{c} P^{-}={\left(p_{1}^{-},p_{2}^{-},\ldots ,p_{m}^{-}\right)}^{T}. \end{array}$$

Then, the value of *p*_*i*_^+^ and *p*_*i*_^−^, respectively, depends on the following situations:

(10) $$\begin{array}{c} p_{i}^{+}=\left\{\begin{array}{cc} \max\left\{b_{ij}^{*}\;|\;j=1,2,\ldots ,n,\;\;i=1,2,\ldots ,m\right\}, & \\ \text{If}\;\text{it}\;\text{is}\;\text{better}\;\text{when}\;\text{the}\;\text{value}\;\text{of}\;b_{ij}^{*}\;\text{is}\;\text{bigger}, & \\ \min\left\{b_{ij}^{*}\;|\;j=1,2,\ldots ,n,\;\;i=1,2,\ldots ,m\right\}, & \\ \text{If}\;\text{it}\;\text{is}\;\text{better}\;\text{when}\;\text{the}\;\text{value}\;\text{of}\;b_{ij}^{*}\;\text{is}\;\text{smaller}, & \\ \overset{n}{\underset{j=1}{\sum }}\frac{b_{ij}^{*}}{n}, & \\ \text{If}\;\text{it}\;\text{is}\;\text{better}\;\text{when}\;\text{the}\;\text{value}\;\text{of}\;b_{ij}^{*}\;\text{is}\;\text{median}, & \end{array}\right. \end{array}$$

(11) $$\begin{array}{c} p_{i}^{-}=\left\{\begin{array}{cc} \max\left\{b_{ij}^{*}\;|\;j=1,2,\ldots ,n,\;\;i=1,2,\ldots ,m\right\}, & \\ \text{If}\;\text{it}\;\text{is}\;\text{better}\;\text{when}\;\text{the}\;\text{value}\;\text{of}\;b_{ij}^{*}\;\text{is}\;\text{bigger}, & \\ \min\left\{b_{ij}^{*}\;|\;j=1,2,\ldots ,n,\;\;i=1,2,\ldots ,m\right\}, & \\ \text{If}\;\text{it}\;\text{is}\;\text{better}\;\text{when}\;\text{the}\;\text{value}\;\text{of}\;b_{ij}^{*}\;\text{is}\;\text{smaller}, & \\ \overset{n}{\underset{j=1}{\sum }}\frac{b_{ij}^{*}}{n}, & \\ \text{If}\;\text{it}\;\text{is}\;\text{better}\;\text{when}\;\text{the}\;\text{value}\;\text{of}\;b_{ij}^{*}\;\text{is}\;\text{median}. & \end{array}\right. \end{array}$$

*Step 5*. Calculate distance between dangerous goods transport enterprise and its ideal points.

Suppose the distance between each enterprise under assessment and its positive ideal point and negative ideal point is *d*_*j*_^+^ and *d*_*j*_^−^, respectively, described as follows:

(12) $$\begin{array}{c} d_{j}^{+}=\sqrt{\overset{m}{\underset{i=1}{\sum }}{\left(b_{ij}^{*}-p_{i}^{+}\right)}^{2}},\;j=1,2,\ldots ,n,\\ d_{j}^{-}=\sqrt{\overset{m}{\underset{i=1}{\sum }}{\left(b_{ij}^{*}-p_{i}^{-}\right)}^{2}},\;j=1,2,\ldots ,n. \end{array}$$

*Step 6*. Calculate closeness between dangerous goods transport enterprise and its ideal points.

The closeness between dangerous goods transport enterprise and ideal points is described as follows:

(13) $$\begin{array}{c} T_{j}=\frac{d_{j}^{-}}{d_{j}^{-}+d_{j}^{+}},\;j=1,2,\ldots ,n. \end{array}$$

*Step 7*. Rank the closeness order of all enterprises according to the value of *T*_*j*_. The bigger the value of *T*_*j*_, the safer the enterprise *j* and less safe in opposite [11].

## 3. The Safety Assessment Optimization Model of Dangerous Goods Transport Enterprise Based on the Relative Entropy Aggregation in Group Decision Making

Through the above-mentioned model of safety assessment of multiobjective dangerous goods transport enterprise based on entropy [12, 13], each expert had calculated the value of *T*_*j*_ for all enterprises in *A*. Suppose *D* = {*d*_*k*_,  *k* = 1,2,…, *q*} is a group decision set, wherein *d*_*k*_ stands for expert *k*. Let *L* = {*l*_*k*_,  *k* = 1,2,…, *q*} be the weight vector of *D*; then use it to reflect the authority that experts have in group decision set, wherein *l*_*k*_ ∈ [0,1], and ∑_*k*=1_^*q*^ *l*_*k*_ = 1; the bigger the value of *l*_*k*_, the more authoritative the expert *k*. The method to calculate weight given by experts [14] is shown as follows.

(1) Generate a set of preferences vector for enterprises *T* = {*T*_*j*_,  *j* = 1,2,…, *n*}, where *T*_*j*_ = {*T*_*kj*_,  *k* = 1,2,…, *q*}, *T*_*j*_ stands for preference vector of the *q* experts preferring enterprise *j*, while *T*_*kj*_ stands for preference of the expert *k* preferring enterprise *j*, and *T*_*kj*_ can be calculated as we described above.
(2) Make a cluster analysis [15] on data *T*_1*j*_, *T*_2*j*_,…, *T*_*qj*_ in *T*_*j*_  (*j* = 1,2,…, *n*). Taking *T*_*j*_, for example, suppose data *T*_1*j*_, *T*_2*j*_,…, *T*_*qj*_ are finally clustered into *x*_*j*_ sorts (*x*_*j*_ ≤ *q*); *y*_*j*_ is the total number in the same sort. Namely, the number of sort 1 is *y*_1*j*_ and *y*_*i*__*j*_ for sort *i*. Let *λ*_*ij*_ be the weight coefficient of expert in sort *i*; then there will be an existing constant *d*_*j*_ which can make formula (14) valid:
  (14) $$\begin{array}{c} y_{ij}=d_{j}\cdot \lambda_{ij}. \end{array}$$
  According to the definition of weight coefficient
  (15) $$\begin{array}{c} \overset{x_{j}}{\underset{i=1}{\sum }}\lambda_{ij}\cdot y_{ij}=1. \end{array}$$
  From formulas (14) and (15) we can know
  (16) $$\begin{array}{c} \lambda_{ij}=\frac{y_{ij}}{\sum_{i=1}^{x}y_{ij}^{2}}. \end{array}$$
(3) Calculate weight coefficient *λ*_*kj*_  (*k* = 1,2,…, *q*, *j* = 1,2,…, *n*) of expert *k* on safety assessment of dangerous goods transport enterprises *j* using the above-mentioned method. Let *λ*_*k*_ = ∑_*j*=1_^*n*^ *λ*_*kj*_  (*k* = 1,2,…, *q*) be the sum of expert *k*'s authority on safety assessment of total *n* dangerous goods transport enterprises.
(4) The weight vector of experts *L* = {*l*_*k*_,  *k* = 1,2,…, *q*} in group decision making can be calculated as the following formula:
  (17) $$\begin{array}{c} l_{k}=\frac{\lambda_{k}}{\sum_{k=1}^{q}\lambda_{k}}. \end{array}$$

When each expert had, respectively, worked out preference on all enterprises in *A* applying the multiobjective decision model based on entropy weight, suppose that the value of *a*_*j*_ can be expressed by cardinal utility and the bigger value indicates that more experts prefer this enterprise, and then we can formalize it as, for all *d*_*k*_ ∈ *D* then there will be a mapping: *π*_*k*_ : *a*_*j*_ → *x*_*kj*_, where *x*_*kj*_ is the value expert *d*_*k*_ assessed on enterprise *a*_*j*_. Let *π*_*g*_ : *a*_*j*_ → *x*_*gj*_ be group preference mapping, and let *X*_*g*_ = (*x*_*g*1_, *x*_*g*2_,…, *x*_*gn*_)^*T*^ be group preference vector; then we can rank the order according to the value of *x*_*gi*_, when we worked out. Subsequently, we can make selection among *A* = {*a*_*j*_,  *j* = 1,2,…, *n*} and compare the preference difference between two enterprises.

The probability measure of preference utility we made on dangerous goods transport enterprises using multiobjective model based on entropy weight is relatively independent discrete random variables; we can also express it in form of consistency preference assessment value using the model combined with relative entropy theory.

Supposing *x*_*i*_, *y*_*i*_ ≥ 0,  *i* = 1,2,…, *n*, and 1 = ∑_*i*=1_^*n*^ *x*_*i*_ ≥ ∑_*i*=1_^*n*^ *y*_*i*_, then we called the following formula the relative entropy *X* referring to *Y*:

(18) $$\begin{array}{c} h\left(X,Y\right)=\overset{n}{\underset{i=1}{\sum }}x_{i}\log\frac{x_{i}}{y_{i}}, \end{array}$$

wherein *X* = (*x*_1_, *x*_2_,…, *x*_*n*_)^*T*^ and *Y* = (*y*_1_, *y*_2_,…, *y*_*n*_)^*T*^.

And *h*(*X*, *Y*) meets the following property if it is relative entropy of *X*, *Y*:

(19) $$\begin{array}{c} \overset{n}{\underset{i=1}{\sum }}x_{i}\log\frac{x_{i}}{y_{i}}=0. \end{array}$$

Only when *x*_*i*_ = *y*_*i*_, *X* and *Y* are two discrete distributions according to the above, the relative entropy can describe correspond degree between.

We can transform the relative entropy model based on group decision making, by minimizing the difference between preference utility value of each expert and preference vector of group, to nonlinear programming problems as follows:

(P) min⁡ QXg=∑k=1qlk∑j=1nlog⁡xgj−log⁡xkj∑j=1nxkjxgjs.t.  ∑j=1nxgj=1, xgj>0.

From formula (P) we can know that preference utility value that each expert made on *A* = {*a*_*j*_,  *j* = 1,2,…, *n*} is limited in interval [0,1] after normalized process. Using the relative entropy theory, we can compare not only the preference utility value of each expert and preference vector of group, but also the preference utility between individuals. Then we discuss the solution of this by generating Lagrange formula and we get the optimal solution *X*_g_^*^ = (*x*_*g*1_^*^, *x*_*g*2_^*^,…, *x*_gn_^*^) shown as follows:

(20) xgj∗=∏k=1qxkj/∑j=1nxkjlk∑j=1n∏k=1qxkj/∑j=1nxkjlk, j=1,2,…,n, k=1,2,…,q.

Rank the order of *A* = {*a*_*j*_,  *j* = 1,2,…, *n*} according to the value of *x*_gj_^*^ in *X*_g_^*^ = (*x*_*g*1_^*^, *x*_*g*2_^*^,…, *x*_gn_^*^) and optimize the selection.

**Figure 1:**
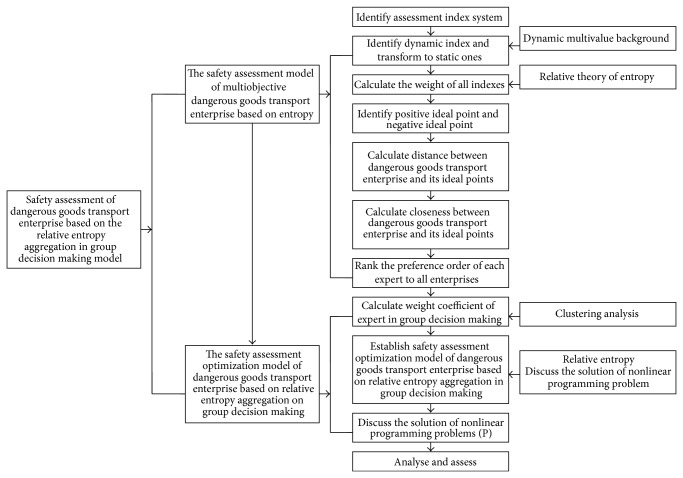
Process of dangerous goods transport enterprise safety evaluation based on relative entropy assembly model in group decision making.

Summing up what we discussed above, we draw the procedure diagram of safety assessment of dangerous goods transport enterprise based on the relative entropy aggregation in group decision making model (see Figure 1).

## 4. Example Research

To improve the safety of road dangerous goods transport in Inner Mongolia, it is necessary to reorganize all the five enterprises in Inner Mongolia. Therefore, we invite three experts (marked by 1, 2, and 3) to make assessment on safety of five enterprises and then reorganize enterprises who have poor safety according to evaluation result [16, 17]. Through amounts of deep survey and analysis, we identified seven safety assessment indexes of dangerous goods transport enterprise as listed below: safety awareness and safe performance skills (b1) of workers, management system of enterprise (b2), safety and operation of facilities (b3), pretransport security check (b4), management and control during transport (b5), prevention measures against damage during transportation of dangerous goods (b6), and mechanism of emergency rescue in safety accident (b7).

*Step 1*. Collect data for above indexes from five enterprises which is going to be assessed. Then make linear transformation on original data, using min-max standardized method, and ensure they are within interval [0,10]. Other indexes, which involve economy, society, and politics and are hard to quantify, come from related professional experts. Those experts rate on satisfaction of indexes according to comprehensive experience and research and the final satisfaction rate within [0,10].

*Step 2*. Identify dynamic indexes and transform to static ones. Because (b1) varies with education degree and work experience, (b6) changes from different goods types and transport route, and (b7) also varies by severity degree of accident, while the left four indexes (b2, b3, b4, b5) are of long-time stability. Now we can easily draw that b1, b6, b7 are dynamic indexes and b2, b3, b4, b5 are static indexes.

Take expert 1, for example, to make a brief description of handling dynamic index statically. Firstly, experts will inspect and analyse dynamic indexes in dangerous goods transport enterprises and rate the satisfaction. Then we get the table of dynamic indexes evaluation of the dangerous goods transport enterprises when *K* = 1 (see Table 1).

Because the attributes of dynamic index are time-varying, the values marked by experts also change at the same time. So we can get Table 2 when *K* = 2.

To simplify example, we consider that the attributes weight of indexes is already known as *u* ^1^ = {0.2,0.1,0.2,0.1,0.1,0.1,0.2} in this paper. Then we can get Table 3 according to formula (3).

*Step 3*. According to the results we got from Steps 1 and 2, and combining with rating of static indexes marked by expert 1, we can get security evaluation value of each index of dangerous goods transport enterprises in Table 4.

*Step 4*. Calculate entropy weight of each dangerous goods transport enterprise. Standardize the matrix which covers all the factors that may affect the safety assessment of dangerous goods transport enterprises and then we can get the standardized matrix *R*. Now we can calculate entropy weight of all factors based on analysis of Table 4; the results are showed as in Table 5.

*Step 5*. Introduce entropy weight into attribute matrix *B*′ and get *B* ^*^; then we can, respectively, work out the positive ideal point and negative ideal point:

(21) $$\begin{array}{c} p_{i}^{+}={\left(0.7408,1.3977,1.3977,1.7415,1.3736,1.1120,0.7408\right)}^{T}\\ p_{i}^{-}={\left(0.5556,0.9318,0.9318,1.1610,1.0302,0.8340,0.5556\right)}^{T}. \end{array}$$

*Step 6*. Calculate closeness of safety of each dangerous goods transport enterprise and rank the order; then we can get the preference order from expert 1 (see Table 6).

Similarly, we can get the reference order from other experts (see Tables 7 and 8).

*Step 7*. Establish optimization model based on the relative entropy aggregation in group decision making, and we can work out the weight factors of experts 1, 2, and 3, respectively, which are 0.32, 0.36, and 0.32. Then we discuss the solution of nonlinear programming problems (P) and we can get the optimal solution as

(22) $$\begin{array}{c} X_{g}^{*}=\left(0.2649,0.2349,0.2090,0.1398,0.1416\right). \end{array}$$

As we know, the value of *x*_gj_^*^ in *X*_g_^*^ reflects the safety level of dangerous goods transport enterprise. The big value for *x*_gj_^*^ indicates the enterprise *j* has more capability to make further development, but not vice versa. Therefore, the final order is as follows: Enterprise 1, Enterprise 2, Enterprise 3, Enterprise 5, and Enterprise 4. So energetic efforts should be put to regulate Enterprise 4.

## 5. Conclusions

Considering the dynamic nature on index value of safety assessment of dangerous goods transport enterprise and nonparity property of weight given by expert, we proposed the safety assessment model of multiobjective dangerous goods transport enterprise based on entropy and the safety assessment optimization model of dangerous goods transport enterprise based on the relative entropy aggregation in group decision making. Then we get the assessment result by discussing the solution. Finally, through assessing the safety of five dangerous goods transport enterprises in Inner Mongolia Autonomous Region, we can see that the improved method we proposed in this paper is practicable and can provide vital decision making basis for reorganizing the dangerous goods transport enterprises.

## Figures and Tables

**Table 1:** Dynamic index evaluation of the dangerous goods transport enterprises when *K* = 1.

Index	Enterprise 1	Enterprise 2	Enterprise 3	Enterprise 4	Enterprise 5
b1	7.8	7.2	8.2	6.8	5.6
b6	8.0	7.9	6.9	6.1	5.9
b7	7.2	7.6	8.0	5.8	7.2

**Table 2:** Dynamic index evaluation of the dangerous goods transport enterprises when *K* = 2.

Index	Enterprise 1	Enterprise 2	Enterprise 3	Enterprise 4	Enterprise 5
b1	8.8	6.2	7.2	7.8	7.6
b6	8.0	8.9	7.9	5.1	6.9
b7	6.2	9.6	8.0	6.8	6.2

**Table 3:** Dynamic index evaluation after static treatment.

Index	Enterprise 1	Enterprise 2	Enterprise 3	Enterprise 4	Enterprise 5
b1	8.0	7.0	8.0	7.0	6.0
b6	8.0	8.0	7.0	6.0	6.0
b7	7.0	8.0	8.0	6.0	7.0

**Table 4:** Each evaluation index value of dangerous goods transport enterprise security evaluation.

Index	Enterprise 1	Enterprise 2	Enterprise 3	Enterprise 4	Enterprise 5
b1	8	7	8	7	6
b2	8	9	7	8	6
b3	9	8	7	6	8
b4	9	9	7	8	6
b5	8	6	6	6	8
b6	8	8	7	6	6
b7	7	8	8	6	7

**Table 5:** Entropy weights of evaluation indexes of dangerous goods transport enterprise security evaluation.

Index	Entropy *H*_*i*_	Entropy weight *λ*_*i*_
b1	0.9966	0.0926
b2	0.9943	0.1553
b3	0.9943	0.1553
b4	0.9929	0.1935
b5	0.9937	0.1717
b6	0.9949	0.1390
b7	0.9966	0.0926

**Table 6:** The order of closeness.

	Enterprise 1	Enterprise 2	Enterprise 3	Enterprise 4	Enterprise 5
Closeness	0.7807	0.5679	0.5018	0.3421	0.3129
Order	1	2	3	4	5

**Table 7:** The order of closeness.

	Enterprise 1	Enterprise 2	Enterprise 3	Enterprise 4	Enterprise 5
Closeness	0.7163	0.6219	0.6918	0.3927	0.4184
Order	1	3	2	5	4

**Table 8:** The order of closeness.

	Enterprise 1	Enterprise 2	Enterprise 3	Enterprise 4	Enterprise 5
Closeness	0.7346	0.7975	0.5716	0.4425	0.4626
Order	2	1	3	5	4

## References

[1] Fabiano B., Currò F., Reverberi A. P., Pastorino R. (2005). Dangerous good transportation by road: from risk analysis to emergency planning. *Journal of Loss Prevention in the Process Industries*.

[2] Xiu L., Zhang L. (2009). Research on road transport of dangerous goods safety management evaluation index system. *Journal of Information Science and Technology*.

[3] Huo Y., He X., Chen J., Li X. (2010). Research on road transport of dangerous goods safety evaluation. *Chinese Journal of Safety Science*.

[4] Yan Y., Liu H., Zhang Y., Chen X., Zhao H. (2010). Safety evaluation method of road transport of dangerous goods enterprises based on fuzzy TOPSIS. *China Safety Science*.

[5] Carotenuto P., Giordani S., Ricciardelli S., Rismondo S. (2007). A tabu search approach for scheduling hazmat shipments. *Computers and Operations Research*.

[6] Gheorghe A. V., Birchmeier J., Vamanu D., Papazoglou I., Kröger W. (2005). Comprehensive risk assessment for rail transportation of dangerous goods: a validated platform for decision support. *Reliability Engineering & System Safety*.

[7] Elshafey M. M., Abd El Halim A. O., Isgor O. B., Contestabile E., Katsabanis T. (2009). Numerical and experimental investigations for safer transportation of dangerous goods. *Journal of Transportation Security*.

[8] Kaye W. E., Orr M. F., Wattigney W. A. (2005). Surveillance of hazardous substance emergency events: identifying areas for public health prevention. *International Journal of Hygiene and Environmental Health*.

[9] Choudhry R. M., Fang D., Mohamed S. (2007). The nature of safety culture: a survey of the state-of-the-art. *Safety Science*.

[10] Xu X., Li C. (2009). Evaluation of train running dynamic index system. *Journal of Railway Transportation and Economy*.

[11] Zhou X., Lu M. (2012). Risk evaluation of dynamic alliance based on fuzzy analytic network process and fuzzy TOPSIS. *Journal of Service Science and Management*.

[12] Ölçer A. I., Odabaşi A. Y. (2005). A new fuzzy multiple attributive group decision making methodology and its application to propulsion/manoeuvring system selection problem. *European Journal of Operational Research*.

[13] Baky I. A. (2014). Interactive TOPSIS algorithms for solving multi-level non-linear multi-objective decision-making problems. *Applied Mathematical Modelling*.

[14] Wibowo S., Deng H. (2013). Consensus-based decision support for multicriteria group decision making. *Computers & Industrial Engineering*.

[15] Trebuňa P., Halčinová J. (2013). Mathematical tools of cluster analysis. *Applied Mathematics*.

[16] Inner Mongolia Department of Statistics (2012). *Inner Mongolia Statistical Yearbook 2003–2013*.

[17] The Inner Mongolia Autonomous Region Transportation Hall (2010). *Highway and Waterway Transportation “Twelfth Five Year Plan”*.

